# A Dosiomics Analysis Based on Linear Energy Transfer and Biological Dose Maps to Predict Local Recurrence in Sacral Chordomas after Carbon-Ion Radiotherapy

**DOI:** 10.3390/cancers15010033

**Published:** 2022-12-21

**Authors:** Letizia Morelli, Giovanni Parrella, Silvia Molinelli, Giuseppe Magro, Simone Annunziata, Andrea Mairani, Agnieszka Chalaszczyk, Maria Rosaria Fiore, Mario Ciocca, Chiara Paganelli, Ester Orlandi, Guido Baroni

**Affiliations:** 1 Department of Electronics, Information and Bioengineering, Politecnico di Milano, Piazza Leonardo da Vinci 32, 20133 Milan, Italy; 2 Medical Physics Unit, National Center of Oncological Hadrontherapy (CNAO), Strada Campeggi, 53, 27100 Pavia, Italy; 3 Heidelberg Ion Beam Therapy Center (HIT), Im Neuenheimer Feld 450, 69120 Heidelberg, Germany; 4 Radiotherapy Unit, National Center of Oncological Hadrontherapy (CNAO), Strada Campeggi, 53, 27100 Pavia, Italy

**Keywords:** dosiomics, outcome prediction, local recurrence, survival analysis, sacral chordoma, LET, RBE modelling

## Abstract

**Simple Summary:**

The poor tumor characterization and the lack of prognostic biomarkers hinder the efficacy and the personalization of treatments for Sacral Chordomas (SC), for which Carbon Ion Radiotherapy (CIRT) is one of the most promising therapeutic options. The aim of this work is to apply, for the first time, a dosiomics approach to biological dose and dose-averaged Linear Energy Transfer (LET_d_) maps, towards the identification of possible prognostic factors and the future integration of decision supportive tools in CIRT workflows. We conducted a time-to-event analysis on a pool of 50 SC patients, investigating the performances of regularized Cox models (r-Cox) and survival Support Vector Machines (s-SVM) in predicting Local Recurrence (LR). LET_d_ distributions confirmed their important role for patient stratification into high/low-risk groups for recurrencies in high-dose regions, showing a potential as a possible source of prognostic factors for CIRT applied to SC.

**Abstract:**

Carbon Ion Radiotherapy (CIRT) is one of the most promising therapeutic options to reduce Local Recurrence (LR) in Sacral Chordomas (SC). The aim of this work is to compare the performances of survival models fed with dosiomics features and conventional DVH metrics extracted from relative biological effectiveness (RBE)-weighted dose (D_RBE_) and dose-averaged Linear Energy Transfer (LET_d_) maps, towards the identification of possible prognostic factors for LR in SC patients treated with CIRT. This retrospective study included 50 patients affected by SC with a focus on patients that presented a relapse in a high-dose region. Survival models were built to predict both LR and High-Dose Local Recurrencies (HD-LR). The models were evaluated through Harrell Concordance Index (C-index) and patients were stratified into high/low-risk groups. Local Recurrence-free Kaplan–Meier curves were estimated and evaluated through log-rank tests. The model with highest performance (median(interquartile-range) C-index of 0.86 (0.22)) was built on features extracted from LET_d_ maps, with D_RBE_ models showing promising but weaker results (C-index of 0.83 (0.21), 0.80 (0.21)). Although the study should be extended to a wider patient population, LET_d_ maps show potential as a prognostic factor for SC HD-LR in CIRT, and dosiomics appears to be the most promising approach against more conventional methods (e.g., DVH-based).

## 1. Introduction

Sacral Chordoma (SC) is a rare, locally aggressive and slow growing malignant tumor that develops at the level of sacrococcygeal region, in close proximity to different organs at risk (OARs, e.g., neurovascular structures connecting to the digestive and reproductive systems). This complex scenario makes the macroscopic surgical resection the most common approach to treat SC, while concurrently being a very delicate and not always possible choice (surgery-unfit patients) that may lead to motor deficits and bowel dysfunction [1]. Therefore, SC treatment typically involves a partial surgical resection followed by adjuvant radiotherapy; however, SC is one of the most challenging tumors to treat because of its critical anatomical location, high tendency to recur, and its recognized resistance to the effects of chemotherapy and radiation [2,3,4,5,6]. Given these limitations, Carbon Ion Radiotherapy (CIRT) has been proposed as a promising alternative for SC treatment, thanks to its high geometrical selectivity and relative biological effectiveness (RBE) with respect to photon radiotherapy (RT), allowing an improved accuracy in the dose delivery and sharper dose gradients [7,8]. Nonetheless, the tendency to recur remains high: given the insidious location of SC, the treatment planning and optimization phase is the most challenging, with the prescribed dose not always being delivered to the Clinical Target Volume (CTV) because of stringent dose constraints on OARs. Moreover, the poor tumor characterization and the lack of prognostic biomarkers do not allow improving the treatment outcome [2]. 

Different approaches have been proposed for the identification of effective signatures and biomarkers that would efficiently help in patient stratification and characterization of tumors, towards a personalized treatment [9,10,11]. Beside patient stratification based on clinical features, the radiomics approach is spreading and gaining interest in the medical field. Radiomics is a machine-learning based method to extract quantitative features from medical images (typically describing shape, intensity, and texture) that can be used to build predictive and prognostic models [12,13,14,15]. Dosiomics represents an extension of radiomics applied to three-dimensional RT dose distributions aiming at extracting useful features for predicting RT treatment outcome [16,17,18]. Indeed, the identification of positive/negative prognostic factors is still an open challenge, especially for complex tumors such as chordomas treated with CIRT. Recently, a dosiomics study applied to skull base chordomas treated with CIRT showed how features describing dose heterogeneity were strongly associated with an adverse outcome [19]. In addition, the dose-averaged Linear Energy Transfer (LET_d_) of carbon ions has been found to correlate with local recurrence in chondrosarcomas [20] and SC [21], increasing interest towards a combined RBE- and LET-based treatment optimization. In this context, dosiomics could be useful to identify possible quantitative prognostic factors, and eventually predict local control to support the treatment planning and optimization. In this regard, the treatment planning phase is still not standardized worldwide, with European facilities exploiting the local effect model version I (LEM I) as the RBE model, while Japanese facilities use the modified microdosimetric kinetic model (mMKM) [22,23]. These models differently correlate with LET_d_ and may offer different performances in terms of local control prediction [21].

Finally, several studies demonstrated that adopting dosiomics features, with their three-dimensional radiation dose information, substantially improve the prediction of RT-induced effects compared with models based on more conventional parameters such as dosimetric parameters derived from the Dose–Volume–Histogram (DVH) [17,24,25,26]. 

This study focuses on dosiomics, investigating the role of biological dose maps (i.e., LEM I- and mMKM-based doses, D_LEM_ and D_MKM_) and LET_d_ distributions as sources of prognostic factors, towards a more efficient and personalized treatment optimization for CIRT applied to SC. To the authors’ best knowledge, no dosiomics study has been conducted on biological dose and LET_d_ maps in SC; this study wants to enlarge and inspect the fields of applicability of dosiomics, towards the identification of prognostic factors for SC treated with CIRT.

## 2. Materials and Methods

### 2.1. Data Collection and Elaboration

Fifty-two patients affected by non-metastatic SC and consecutively treated with CIRT at the National Centre for Oncological Hadrontherapy (CNAO) between 2013 and 2018 were retrospectively analyzed. All the patients were treated with CIRT after macroscopical surgical resection or biopsy alone. None of these received adjuvant conventional RT. Inclusion criteria were: (i) a prescription dose of 70.4 Gy (RBE) or 73.6 Gy (RBE) delivered in 16 fractions, following a sequential boost scheme with target shrinkage after 9 fractions; (ii) any surgical resection degree (macroscopic complete or only biopsy); (iii) a 12-months minimum follow-up; (iv) the availability of complete clinical and dosimetric data. Detailed characteristics of the patient cohort are reported in supplementary materials Section S1.

Treatment plans were optimized with the Syngo RT planning (Siemens AG Healthcare, Erlangen, Germany) treatment planning system (TPS) based on the LEM I radiobiological model, with α/β = 2 Gy. The dose was prescribed to the Low-Dose Clinical Target Volume (CTV_LD_) for the first 9 fractions, and on the High-Dose CTV (CTV_HD_) for the following 7.

Starting from the 3D physical dose distribution maps, D_LEM_ and D_MKM_ biological dose maps were calculated using LEM-I and mMKM models, respectively. In addition, maps of LET distribution were calculated for each patient. All the calculations were performed using FRoG, a GPU-based dose evaluation engine [27]. D_LEM_ and D_MKM_ maps from the 9 and 7 fractions were summed to obtain the overall biological doses, while LET maps were combined into a dose-averaged LET map (LET_d_) following the approach suggested by Matsumoto et al. [20].

CTV_HD_ contours, manually delineated on treatment planning CT scan, were collected as volume of interest (VOI) for this work, being the target volume of the whole treatment (9+7 fractions) [28,29].

The treatment outcome was recorded at follow-up in terms of local control (LC) and local recurrence (LR), classifying a local disease-free survival as LC (favorable event), while a recurrence or disease progression in the target volume (clinically assessed on radiological imaging) as LR (adverse event). After a median follow-up time of 42.6 months, a LR was found in 26 patients (52%, median time-to-recurrence = 29.2 months), while 24 patients (48%) were included in the LC group (median follow-up time = 36.7 months). 

Among LR patients, two radiation oncologists and a medical physicist classified the recurrencies as “in-field”, “field-edge” or “out-of-field”, depending on the location of occurrence with respect to the dose coverage [21]: LR patients (26) included 2 field-edge and 24 in-field recurrencies. Moreover, the in-field recurrencies were classified as High dose (HD-LR) or Low-dose (LD-LR), depending on the dose coverage on the site of recurrence: HD-LR were located in a region of acceptable dose coverage (CTV_HD_ D_LEM|95%_ ≥ 95% of the prescribed dose), while LD-LR were placed in an underdosed region (CTV_HD_ D_LEM|95%_ < 95% of the prescribed dose), respectively. In this context, 13 in field recurrencies were classified as HD-LR, while 11 as LD-LR because of a suboptimal dose coverage of the target due to OARs constraints [21]. 

The study was approved by the local Ethical Review Board and the informed consent (CNAO OSS 24/2021) signed by all patients. The dataset was the same as that used by Molinelli et al. [21].

### 2.2. Feature Extraction and Selection

**Figure 1:**
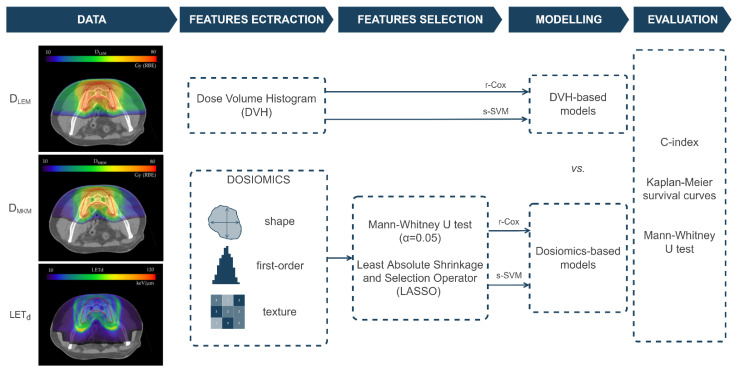
The proposed workflow. Dosimetric parameters and dosiomics features were extracted from D_LEM_, D_MKM_ and LET_d_ maps. Mann–Whitney U test (MW) and Least Absolute Shrinkage and Selection Operator (LASSO) were used as selection routines for dosiomics features. Survival models based on r-Cox and s-SVM were both used to build DVH-based and dosiomics-based models for LR and HD-LR prediction. A stratified 5-fold cross-validation was performed to evaluate the models through C-index, Kaplan–Meier survival curves and Mann–Whitney U test.

A total of 107 dosiomics features (14 shape, 18 first-order and 75 texture features) were extracted from D_LEM_, D_MKM_ and LET_d_ maps (Figure 1) using PyRadiomics (v3.0.1), following the guidelines of the Image Biomarker Standardization Initiative [9]. The features were extracted in 3D from the original maps, without applying filters or normalization or resampling. Texture features were extracted from gray level co-occurrence matrix (GLCM, *n* = 24), gray level run length matrix (GLRLM, *n* = 16), gray level size zone matrix (GLSZM, *n* = 16), gray level dependence matrix (GLDM, *n* = 14), and neighboring gray tone difference matrix (NGTDM, *n* = 5). Details of the dosiomics features used in this study are reported in supplementary materials Section S2. 

Feature extraction was performed with a specific bin width for each map. Specifically, the Freedman–Diaconis rule [30,31] was applied to find, for each map, the optimal bin width for its specific distribution of values in the histogram. Therefore, separately for each map, the median value of the optimal bin width calculated among all patients was chosen, which was found to be 0.10, 0.25 and 0.50 for D_LEM_, D_MKM_ and LET_d_, respectively.

Two different feature selection methods were compared and applied on dosiomics features, to reduce data redundance and avoid overfitting (Figure 1): (i) Mann–Whitney U test, and (ii) least absolute shrinkage and selection operator (LASSO) were used (independently) to select the most useful features. Specifically, the Mann–Whitney U test was applied to select statistically significant features with a *p*-value < 0.05 (MW-routine, [32,33,34]). In addition, to decrease the high dimensionality of the dosiomics features, LASSO regression, an effective dimensionality reduction method, was applied (encapsulated in a repeated 5-fold cross-validation routine), and the 10 best features were selected for each map (LASSO-routine, [25,32,34,35]).

In addition to the dosiomics features, dosimetric parameters extracted from the DVH were considered, in order to compare the performance of dosiomics models with those obtained with more conventional parameters. Similarly to Molinelli et al. [21], D95 (i.e., the highest dose received by 95% of the object volume), D50, D2 and structure volume (i.e., CTV_HD_) were extracted from the D_LEM_ and D_MKM_ maps. As suggested by Matsumoto et al. [8], the dosimetric parameters extracted on LET_d_ maps were the volume receiving at least 50 keV/µm (V_50keV/µm_), the LET_d_ given to 1 mL of CTV_HD_ (L_1mL_), the median LET_d_ (LET_d|50%_) and the structure volume. Dosimetric features did not undergo any feature selection. 

### 2.3. Model Building

In order to investigate and compare the prognostic power of dosiomics features and dosimetric parameters, survival models based on (i) linear survival support vector machine (s-SVM, scikit-survival v.0.18) and (ii) conventional Cox proportional hazard model regularized with an elastic-net penalty (r-Cox, scikit-survival v.0.18) were considered.

Before model building, dosiomics features and dosimetric parameters were normalized using z-score and L2-norm for s-SVM and r-Cox, respectively. Survival models were then independently built from the selected dosiomics features (i.e., dosiomics-based models) and the dosimetric parameters extracted from the DVH (i.e., DVH-based models) investigating the ability to correctly discriminate patients with LC (*n* = 24) from those with LR (*n* = 26). In addition, in agreement with Molinelli et al. [21] who preliminarily investigated the differences between LC and HD-LR (*n* = 13) patients, LC vs. HD-LR survival models were built. Indeed, Molinelli et al. [21] showed interest in investigating treatment failure in patients with HD-LR as they presented a relapse in a high-dose region. Differently, for patients with LD-LR, the relapse was attributable to an underdosage due to proximity to OARs. As such, the two architectures (i.e., s-SVM and r-Cox) were both employed to build dosiomics-based and DVH-based models aiming at assessing their performance in predicting LR and HD-LR.

In the model building phase, the hyper-parameters were tuned through a cross-validated (repeated 5-fold cross-validation) grid-search, by maximizing the Harrell Concordance index (C-index). Specifically, the regularization parameter alpha, the rank ratio and the optimizer were tuned for s-SVM, while the l1-ratio and the penalty factors for r-Cox (supplementary materials Section S3, Table S2).

### 2.4. Model Evaluation

In order to evaluate and compare the prognostic power of the dosiomics-based and DVH-based models, a stratified 5-fold cross-validation routine was randomly repeated 10 times, obtaining 50 different survival models for each setting (e.g., dosiomics-based s-SVM model for LR prediction) producing 10 outcome predictions for each patient. Specifically, the outcome prediction and thus the stratification of each patient into low/high risk was obtained starting from the raw output of the model by setting the stratification cut-off to the median value of the model’s output on the training set. Then, to evaluate the performance of the model downstream of the repeated cross-validation, the 10 outcome predictions of each patient were pooled together through majority-voting to obtain a single low/high risk prediction for each patient. From the low/high-risk stratifications thus obtained, Kaplan–Meier (KM) survival curves were estimated, investigating the usefulness of this approach for predicting the recurrence-free survival probability.

Finally, the performance of the constructed models was quantified: (i) the median value and the interquartile range (IQR) of the C-indices were calculated to evaluate the performance of repeated cross-validations, (ii) Mann–Whitney U test (α = 0.05) was applied on parameters and features between the low- and high-risk classes, defined according to the model output, (iii) log-rank tests (α = 0.05) were performed between the Kaplan–Meier estimated survival functions.

## 3. Results

The r-Cox architecture outperformed the s-SVM in predicting both outcomes (i.e., LR and HD-LR), for both types of models (i.e., DVH-based and dosiomics-based). Therefore, in this section the results related to r-Cox will be presented, while those obtained with s-SVM are reported in supplementary materials Section S4 (Table S3).

### 3.1. Dosiomics-Based Models Predicting Overall Recurrence

The Mann–Whitney U test (MW) was applied to select dosiomics features with statistically significant differences between LC and LR patients. Specifically, three shape features (i.e., Elongation, Flatness and Sphericity) were found to be statistically significant (lower values for LR patients). By definition, shape features are independent from the map content but only depend on the volume on which they are calculated (i.e., CTV_HD_). Thus, since they are the same regardless of which map we consider, they were selected by the significance test (i.e., MW-routine) for all the maps. In addition to shape features, First-Order-Kurtosis and First-Order-Skewness were selected for LET_d_, while the GLCM-Correlation for D_MKM_. Differently, no significant features were found in the D_LEM_ except for the three shape features. The features set obtained with MW was therefore considered null for D_LEM_ (i.e., n.a. in Table 1), since a shape-only set would not be representative of the performance of D_LEM_ map content, but instead of the CTV_HD_ contours. The median values of the C-indices obtained on the models built from the MW-routine were 0.71 and 0.70 for LET_d_ and D_MKM_, respectively (Table 1, top-left). Statistically significant differences were also found in features between low- and high-risk patients as stratified downstream of the r-Cox survival model for LET_d_ and D_MKM_ (supplementary materials Section S5, Figure S1). However, no significant differences emerged between the low- and high-risk Kaplan–Meier curves, with *p*-values of 0.0937 (LET_d_) and 0.1826 (D_MKM_) (Figure S3). For results related to models built including shape features only, please refer to supplementary materials Section S6.

**Figure 2:**
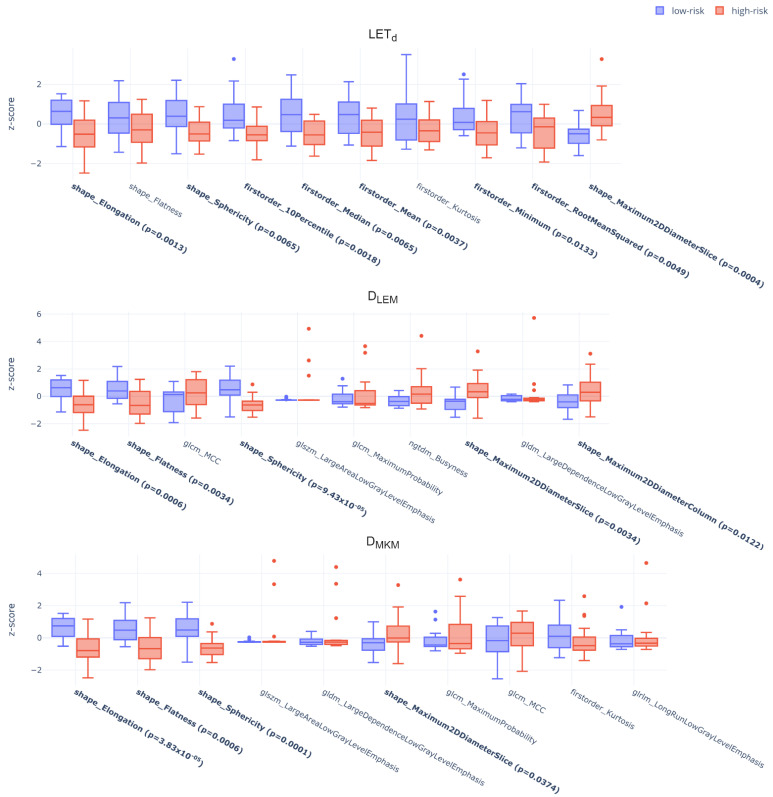
Standardized dosiomics features (LASSO-routine, LET_d_, D_LEM_, D_MKM_ from top to bottom) as stratified by r-Cox according to the risk (low-risk in blue, high-risk in red) of LR. Features highlighted in bold showed statistically significant differences between the two classes (Mann–Whitney U test (α = 0.05)) and the obtained *p*-values are reported in brackets. Each label in the boxplot is structured as feature type (i.e., ‘shape’ for shape features, ‘firstorder’ for first order features and matrix name (e.g., ‘glcm’) for texture features) followed by the specific feature name according to PyRadiomics convention. Refer to supplementary materials Section S2 for more details.

**Figure 3:**
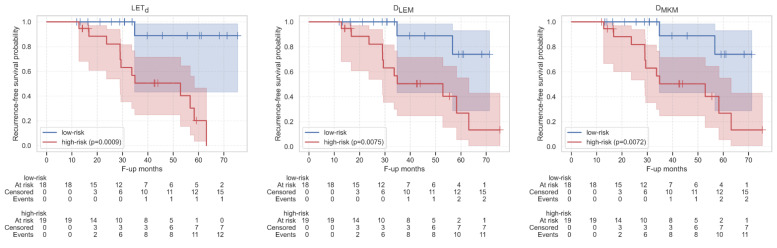
Kaplan–Meier survival curves for patients at high-(red) and low-risk (blue) of an LR as stratified by r-Cox using LET_d_ (**left**) D_LEM_ (**middle**), D_MKM_ (**right**) features selected by LASSO. Shaded areas show curves confidence intervals and the *p*-values obtained from the comparison between high- and low- risk patients are reported in the legend. Censored events are highlighted with the symbol ‘+’. Below the plot, the number of patients belonging to each risk group at specific time points (months) is reported.

Concerning LASSO feature selection, the 10 best features were selected for each map (supplementary material, Figure S2). Among the survival models built from these feature sets, the LET_d_ map achieved the highest C-index (0.71), against D_LEM_ and D_MKM_ (0.70 and 0.69, respectively, Table 1, top-left). However, only these two maps resulted in a significant patient stratification on Kaplan–Meier curves: the *p*-values were 0.0553, 0.0201 and 0.0111 for LET_d_, D_LEM_ and D_MKM_, respectively. Figure 2 shows standardized dosiomics features between patients at low- (blue) or high-risk (red) of developing a LR are reported for LET_d_, D_LEM_, D_MKM_ models. Specifically, stratifications obtained from all three maps showed that high risk of LR was associated with lower values of shape Elongation, Flatness, and Sphericity. In addition, from the stratification obtained from the LET_d_ (Figure 2, top), high-risk patients were associated with lower values of first-order Kurtosis, Skewness and Minimum. Finally, from the stratification obtained with D_LEM_, higher values of GLCM Correlation and MCC (i.e., Maximal Correlation Coefficient) were associated with high-risk of LR (Figure 2, middle). Kaplan–Meier survival curves estimated for LET_d_, D_LEM_ and D_MKM_ from this stratification are reported in Figure 3.

### 3.2. Dosiomics-Based Models Predicting in-Field Recurrence

When investigating the prognostic power in differentiating LC from HD-LR patients, the MW selection routine allowed the identification of 5, 4 and 10 significant features in LET_d_, D_LEM_ and D_MKM_, respectively, and, among these, only one shape feature was included (i.e., Elongation) in all the sets (supplementary materials, Figure S6 top-row). The median C-index values were 0.80 (LET_d_), 0.80 (D_LEM_) and 0.76 (D_MKM_). However, when feature differences between the risk groups as stratified by the survival model were investigated, except for shape Elongation, no significant differences were found in any map (supplementary materials, Figure S6 bottom-row). The survival curves analyses, instead, showed a significant difference between the two stratified groups for LET_d_ (*p* = 0.0060) and D_LEM_ (*p* = 0.0174) but not for D_MKM_ (*p* = 0.1076, supplementary materials Figure S7).

**Figure 4:**
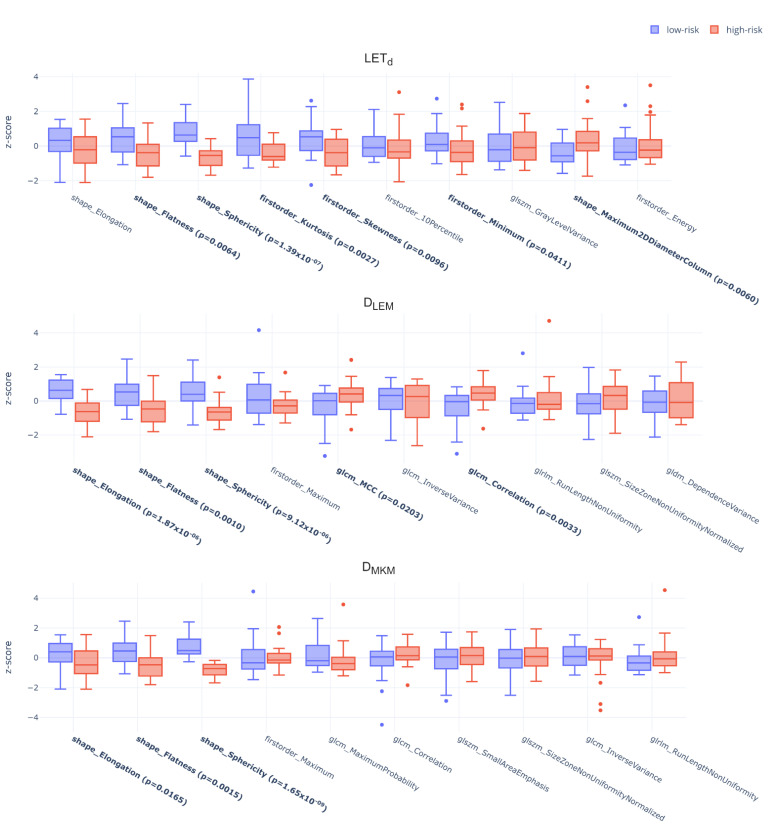
Standardized dosiomics features (LASSO-routine, LET_d_, D_LEM_, D_MKM_ from top to bottom) as stratified by r-Cox according to the risk (low-risk in blue, high-risk in red) of HD-LR. Features highlighted in bold showed statistically significant differences between the two classes (Mann–Whitney U test (α = 0.05)) and the obtained *p*-values are reported in brackets. Each label in the boxplot is structured as feature type (i.e., ‘shape’ for shape features, ‘firstorder’ for first order features and matrix name (e.g., ‘glcm’) for texture features) followed by the specific feature name according to PyRadiomics convention. Refer to supplementary materials Section S2 for more details.

**Figure 5:**
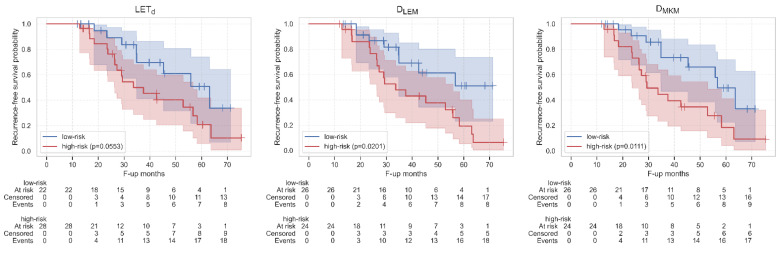
Kaplan–Meier survival curves for patients at high-(red) and low-risk (blue) of HD-LR as stratified by r-Cox using LET_d_ (**left**) D_LEM_ (**middle**), D_MKM_ (**right**) features selected by LASSO. Shaded areas show curves confidence intervals and the *p*-values obtained from the comparison between high- and low-risk patients are reported in the legend. Censored events are highlighted with the symbol ‘+’. Below the plot, the number of patients belonging to each risk group at specific time points (months) is reported.

With the LASSO feature selection, the 10 best features for predicting HD-LR were selected for each map (supplementary materials, Figure S8) and the analyses showed that these LASSO-derived sets achieved peak performance for all maps, yielding a median C-index of 0.86, 0.83 and 0.80 for LET_d_, D_LEM_ and D_MKM_, respectively. Statistically significant differences were also found in features between low- and high-risk patients as stratified downstream of the survival model for all the maps, but, unlike LET_d_, in D_LEM_ and D_MKM_ these were only shape features (Figure 4). Specifically, from the LET_d_ distribution, statistically significant differences between the two classes of estimated risk were found for the first-order features Median, Mean, Root Mean Squared, 10Percentile and Minimum. The first three were also statistically different in the two classes of treatment outcome in input to the model (LC and HD-LR, supplementary material Figure S8, top), while the 10Percentile and Minimum, despite showing the same trend, became significant only downstream of the survival model (Figure 4, top). In addition, also the survival curves achieved peak performance, showing significant differences in estimated probabilities between the two risk groups, with *p*-values of 0.0009, 0.0075 and 0.0072 for LET_d_, D_LEM_ and D_MKM_ (Figure 5).

### 3.3. DVH-Based Models

In case of LC vs. LR, the DVH-based models trained with dosimetric parameters (supplementary materials, Figure S4) achieved lower performance in terms of both C-index (Table 1, top-right) and survival curves separation (with *p*-values always above the significance threshold, supplementary materials, Figure S5) than dosiomics models. In particular, the highest C-index was obtained with LET_d_ reaching a maximum of 0.58 (0.45 was instead obtained with both D_LEM_ and D_MKM_).

Similarly, even in the case of HD-LR prediction, the dosimetric parameters employed to build the DVH-based models (supplementary materials, Figure S9) achieved a worst performance than dosiomics models, with C-indices of 0.61 (LET_d_), 0.65 (D_LEM_) and 0.64 (D_MKM_). Kaplan–Meier curves for all maps also showed no statistically significant differences in recurrence-free survival probabilities between low- and high-risk patients (supplementary materials, Figure S10).

## 4. Discussion

With this work we aimed at training two survival models (i.e., r-Cox and s-SVM) with dosiomics features and DVH-based parameters extracted from RBE-weighted dose (i.e., D_LEM_ and D_MKM_) and LET_d_ maps, with the final goal of predicting LR for patients affected by SC and treated with CIRT, stratifying them in high- and low-risk groups. The models, tuned through a repeated 5-fold cross-validation, were evaluated through C-index, log-rank test on KM curves and MW test downstream of the stratification. In particular, we investigated those cases affected by local relapses in a region of high dose (i.e., HD-LR), to exploit potential prognostic factors for a better characterization of the treatment in those regions. Low-dose relapsed cases (LD-LR) instead were not singularly investigated because the region of interest did not receive the necessary prescribed dose because of constraints on OARs, thus affecting the treatment outcome [21].

In general, r-Cox models trained with dosiomics features to predict HD-LR always resulted in an improved performance with respect to those predicting LR. This could be related to the fact that the sub-optimal dose distribution in LD-LR patients may have introduced a bias in the LR class in which they were included (LR = HD-LR + LD-LR), hindering the actual association between prognostic characteristics present in dose distributions and the probability of LR. 

### 4.1. Dosiomics-Based Models Predicting Overall Recurrence

Elongation, Sphericity and Flatness were selected both from MW- and LASSO-routine for LC vs. LR patients, suggesting that the shape of the CTV_HD_ was significantly different in the two groups, with a more irregular and flatter shape in LR patients. This is in accordance with most of literature studies that present the volume and shape of the lesion to be some of the most relevant negative prognostic factors [7,36,37]. 

In addition to these common features, MW-routine highlighted first-order Skewness and Kurtosis on LET_d_ maps as relevant and statistically different features between LC and LR. This observation suggests that the LC group described a distribution of LET_d_ values concentrated towards a spike close to the mean value and with a longer tail on high LET_d_ values, while the distribution was wider and values were more spread along the tails in the case of LR patients. These two features were selected also by the LASSO-routine and kept their significance also in the high/low-risk stratification, suggesting a possible role in risk assessment. Specifically, these results seem to suggest that, although LET_d_ values are on average comparable inside the CTV_HD_ (i.e., non-significant differences in mean/median/maximum values), a more heterogeneous distribution of LET_d_ values within the target is associated with a higher probability of a local treatment failure (i.e., LR). However, these considerations should be evaluated in light of the small dataset under analysis (i.e., 50 patients), which is not completely representative of the population, and does not allow us to draw firm conclusions. In this context, survival curves were not statistically significant (Figure 2, left), weakening the overall performance of LET_d_ maps for LR prediction. Similarly, the D_MKM_-based model built with significative features only could not significantly stratify the two risk groups (Figure 2, right), possibly because of the low number of features considered.

On the other hand, the results obtained with the D_LEM_-based model built with LASSO-selected features showed patients at high-risk of LR to be associated with high values of GLCM Correlation and MCC, both indicating the presence of periodic patterns within the dose map. The values of these features, however, were not statistically different between the two classes in input to the model (i.e., LC ad LR, supplementary materials, Figure S2, middle), becoming significant only when compared between the two risk output classes. Since the estimated risk classes were statistically different in terms of recurrence-free survival probability (*p* = 0.0201, Figure 2), this can suggest a potential prognostic role for these texture features, which from our analyses, can be considered as the most promising in the LR prediction. Indeed, despite all dosiomics models showing comparable results among them in terms of C-index (i.e., C-indices were, 0.71, 0.70, 0.69 for LET_d_, D_LEM_, D_MKM_, respectively), only D_LEM_- and D_MKM_-based models described significantly different KM curves (*p*-values were 0.02 and 0.01, respectively), with D_LEM_ maps being the only showing texture features with relevant roles. This, however, seems a reasonable result since all patients were treated with a plan optimized with a LEM-I RBE model.

### 4.2. Dosiomics-Based Models Predicting in-Field Recurrence

The promising results obtained for LR analyses were outperformed by the models predicting HD-LR, both in terms of median C-index, with the peak values of 0.86, 0.83 and 0.80 for LET_d_-, D_LEM_- and D_MKM_-based models, and in terms of KM curves.

In this case, MW-routine selected multiple features including shape, first-order, and texture (supplementary materials, Figure S6, top). Considering this restricted dataset (i.e., LC vs. HD-LR), Elongation was the only significant shape feature between the two groups, indicating a more irregular and non-spherical shape of the lesion in HD-LR patients. This feature was always selected also from LASSO-routine, and significantly separated downstream patient stratification. This confirms what was also found in LC vs. LR analyses and is in agreement with most literature studies reporting tumor volume and shape as some of the most relevant negative prognostic factors [7,36,37].

Concerning LET_d_ maps, LC patients were characterized by a significative higher Mean, Median, Root Mean Squared and 90Percentile (supplementary materials, Figure S6, top-left), along with the findings by Molinelli et al. [21] who recognized a significantly lower median LET_d_ in HD-LR patients. 

Texture features were instead selected in D_LEM_ and D_MKM_ biological maps: GLDM Gray Level Variance, along with the first-order Mean Absolute Deviation and Variance in D_LEM_ (supplementary materials, Figure S6, top-center), while nine significant first-order and texture features in D_MKM_ maps (supplementary materials, Figure S6, top-right). However, comparing each map, of the MW-selected features downstream patient stratification in high- and low-risk, none were shown to be statistically significant except shape Elongation, suggesting that the patient stratification in MW-based models was mainly guided by shape features, while first-order and texture ones did not have a strong impact. In support of this consideration and to better quantify the impact of shape features on the performance, we decided to add in supplementary materials Section S6 (Figures S11 and S12) evaluations on a model built with only shape features. 

In line with this, LASSO did not select any other statistically significant feature except for Elongation in D_LEM_ and D_MKM_ maps, while all the significant features (i.e., Median, Mean and Root Mean Squared, supplementary materials Figure S8) were picked in the case of LET_d_ maps. In this context, the LET_d_-based model reached the peak performance, with a median C-index of 0.86 and KM curves highly separated (*p* = 0.0009). These significant features were found to be statistically significant even downstream of the stratification (Figure 4, top). In addition, patients at high-risk of HD-LR were significantly associated also with lower values of 10Percentile and Minimum (Figure 4, top). 

Overall, the excellent stratification in low/high-risk obtained with the LASSO-selected features set leads to the conclusion that lower values of LET_d_ within the CTV_HD_ are highly predictive of an in-field local recurrence (i.e., HD-LR). 

In contrast, similarly to what was achieved for MW-based models for HD-LR, the performance of D_LEM_ and D_MKM_, although optimal, were mainly driven by the information of shape features (Figure 4), weakening the actual contribution of the maps content itself. While in the case of LR the D_LEM_ was found to be the most predictive, this decreased its prognostic abilities in discriminating LC patients from those with HD-LR in favor of LET_d_ map, which emerged to be the most informative one for predicting HD-LR. 

Overall, we assume that the ability of the D_LEM_ map in predicting LR was probably influenced by the presence of patients with a sub-optimal LEM-based plan (i.e., underdosage of the CTV_HD_ on 13 out of 26 relapsed patients). Nevertheless, results in HD-LR (i.e., patients that received an optimal dose coverage) do not include any bias due to the presence of recurrencies in low-dose regions and confirmed that LET_d_ maps can be considered as a promising source of prognostic factors.

### 4.3. DVH-Based Models and General Considerations

Finally, DVH-based models could not efficiently discriminate the two groups of patients, with a best-case C-index close to randomness for LET_d_-, D_LEM_- D_MKM_-based models and none of the KM curves being significantly discerned (*p*-value > 0.05), thus attesting the potential of a dosiomics analysis for LR and HD-LR prediction. Indeed, both the promising C-indexes and the significative separation of KM curves in most cases support the improved performance of the dosiomics features against conventional DVH metrics in accordance with the literature [17,24,25,26], with LET_d_-based model being the most predictive. This also supports the generalization of our procedure as LET_d_ does not strongly depend on radiobiological models adopted at different facilities, as for biological doses. In addition, the most predictive LET_d_ features were those of the first-order type: this further strengthens the generalization of these potential predictors that do not depend on the choice of the discretization parameters, resulting more robust and repeatable than texture features [38,39,40]. Nonetheless, the limited dataset available and the lack of a test set represent two major limitations of this study and hinder its generalization. In addition, the limited dataset employed for this work did not allow a detailed analysis on the reproducibility of features, the segmentation routine and other factors that influence the extraction of dosiomics features [38,39,40,41]. Moreover, considering the slow-growth progression of chordomas, the follow-up time of the patient cohort is relatively short: on average it is shorter in the control group (i.e., 37 months, supplementary materials Table S1) than in the relapsed group (i.e., 49 months), probably affecting the results. However, since the median time-to-recurrence (i.e., 29 months) was below the median follow-up times of both the groups, the follow-up time is probably appropriate and sufficient for the patient cohort under investigation. Nevertheless, the possibility of extending these analyses to longer follow-up times would allow for more robust and generalizable results.Despite these limitations, we believe that these results are promising and put forward to further studies on the application of dosiomics to CIRT.

## 5. Conclusions

LET_d_, D_LEM_ and D_MKM_ maps were, for the first time, integrated into a dosiomics-based time-to-event analysis to predict the risk of developing recurrence in SCs treated with CIRT. D_LEM_ maps showed to be the most promising source of possible prognostic factors in the case of local recurrence (LR), but the presence of recurrencies due to an underdosage of the target could have affected the results. Nevertheless, the analysis performed on a subset of patients (i.e., HD-LR) were instead more representative, with LET_d_ maps leading to the best performance. Although further analysis is needed, the dosiomics features extracted from the maps, and in particular from LET_d_, showed very promising results pushing towards the identification of possible prognostic factors for SC treated with CIRT. 

## Figures and Tables

**Table 1:** C-indices corresponding to the r-Cox models with different settings. Results are shown in terms of median/IQR. Best values for each map are highlighted with the symbol *.

		Dosiomics-Based			DVH-Based		
	Selection	LET_d_	D_LEM_	D_MKM_	LET_d_	D_LEM_	D_MKM_
LC vs. LR	MW	0.71/0.19	n.a.	0.70/0.19	0.58/0.29	0.45/0.12	0.45/0.17
	LASSO	0.71/0.18	0.70/0.18	0.69/0.15			
LC vs. HD-LR	MW	0.80/0.21	0.80/0.22	0.76/0.32	0.61/0.18	0.65/0.38	0.64/0.39
	LASSO	0.86/0.22 *	0.83/0.22 *	0.80/0.21 *			

## Data Availability

The data presented in this study are available upon reasonable request.

## Supplementary Materials

The following supporting information can be downloaded at: https://www.mdpi.com/article/10.3390/cancers15010033/s1, Figure S1: Z-standardized features as stratified according to the risk of showing a LR. The models are built with dosiomics features from LET_d_ and D_MKM_, after MW feature selection; Figure S2: Z-standardized features as stratified according to the of showing a LR. The models are built with dosiomics features from LET_d_, D_LEM_ and D_MKM_ maps after LASSO feature selection; Figure S3: Kaplan-Meier survival curves for patients at high- and low-risk of a LR as stratified by r-Cox using LET_d_ and D_MKM_ features after MW selection; Figure S4: Z-standardized features as stratified according to the risk of showing a LR. The models are built with dosimetric features from LETd, D_LEM_ and D_MKM_ maps; Figure S5: Kaplan-Meier survival curves for patients at high- and low-risk of a LR as stratified by r-Cox using LET_d_, D_LEM_ and D_MKM_ dosimetric features; Figure S6: Z-standardized features as stratified according to the real class and the risk of showing a HD-LR. The models are built with dosiomics features from LET_d_, D_LEM_ and D_MKM_ maps, after MW feature selection; Figure S7: Kaplan-Meier survival curves for patients at high- and low-risk of a HD-LR as stratified by r-Cox using LET_d_, D_LEM_ and D_MKM_ features after MW selection; Figure S8: Z-standardized features as stratified according to the risk of showing HD-LR. The models are built with features from LET_d_, D_LEM_ and D_MKM_ maps, after LASSO feature selection; Figure S9: Z-standardized features as stratified according to the of showing a HD-LR. The models are built with dosimetric features from LET_d_, D_LEM_ and D_MKM_ maps; Figure S10: Kaplan-Meier survival curves for patients at high- and low-risk of a HD-LR as stratified by r-Cox using LET_d_, D_LEM_ and D_MKM_ dosimetric features. Figure S11: Z-standardized features as stratified according to the risk of showing a LR or HD-LR. The models are built with LASSO-selected shape features. Figure S12: Kaplan-Meier survival curves for patients at high- and low-risk of a LR or HD-LR as stratified by r-Cox using LASSO-selected shape features; Table S1: Relevant clinical information on relapsed and control patients cohorts; Table S2: Possible values for each Hyperparameter during grid-search optimization; Table S3: Results of the s-SVM models with different settings.
