# Supplementary material for: A Dosiomics Analysis Based on Linear Energy Transfer and Biological Dose Maps to Predict Local Recurrence in Sacral Chordomas after Carbon-Ion Radiotherapy
Source: Cancers (Basel). 2022 Dec 21;15(1):33. doi: 10.3390/cancers15010033 (PMC9817801; doi:10.3390/cancers15010033)
Supplement: Supplementary file 1 [file cancers-15-00033-s001.zip › cancers-2069851-supplementary.pdf]

cancers

MDPI

Article

Supplementary materials: A dosiomics analysis based on linear energy transfer and biological dose maps to predict Local Recurrence in Sacral Chordomas after Carbon-Ion Radiotherapy

Letizia Morelli and Giovanni Parrella, Silvia Molinelli, Giuseppe Magro, Simone Annunziata, Andrea Mairani, Agnieszka Chalaszczyk, Maria Rosaria Fiore, Mario Ciocca, Chiara Paganelli, Ester Orlandi and Guido Baroni

Section S1: Patient data

50 SC patients were retrospectively selected for the study according to the following inclusion and exclusion criteria:

- *Inclusion criteria*
    1. prescription dose of 70.4 Gy (RBE) or 73.6 Gy (RBE) delivered in 16 fractions, following a sequential boost scheme with target shrinkage after 9 fractions
    2. any surgical resection degree (macroscopic complete or only biopsy)
    3. 12-months minimum follow-up
    4. availability of complete clinical and dosimetric data
- *Exclusion criteria*
    1. metastatic disease
    2. no histological diagnosis
    3. previous radiotherapy in affected region
    4. concomitant chemotherapy
    5. extensive metal instrumentation/implants
    6. inability to hold the treatment positioning
    7. pregnancy

Detailed information on the patient cohort are reported in Table S0.

Table S1. Relevant clinical information on relapsed and control patients cohorts.

	**Relapsed**	**Control**
Number of patients	26	24
	Value (patients)	*Value (patients)*
Prescribed dose [Gy(RBE)]	70.4 (19) - 73.6 (7)	70.4 (17) - 73.6 (7)
Number of fields	2 (17) - 3 (9)	2 (15) - 3 (9)
	Median (range)	*Median (range)*
Follow-up time [months]	49 (17-86)	37 (12-75)
Time-to-recurrence [months]	29 (13-64)	-
GTV [cc]	337 (2-1738)	466 (22-2678)
CTV_{HD} [cc]	844 (104-2397)	1145 (89-4351)
CTV_{LD} [cc]	1621 (286-3411)	1883 (182-4714)

Section S2: Features classes

Features were extracted from raw dose (i.e., LET-I and mMKM) and LET_d maps, through PyRadiomics modules (v. 3.0.1). For a complete description of each feature, please refer to PyRadiomics official documentation. Features were extracted from 3D patches, containing the following classes:

- **Shape**

Elongation, flatness, least axis length, major axis length, maximum 2D diameter column, maximum 2D diameter row, maximum 2D diameter slice, maximum 3D diameter, mesh volume, minor axis length, sphericity, surface area, surface volume ratio, voxel volume.

- **First-order**

10th percentile, 90th percentile, energy, entropy, interquartile range, total energy, kurtosis, maximum, mean absolute deviation, minimum, range, mean, median, robust mean absolute deviation, root mean squared, skewness, uniformity, variance.

- **Texture:**

  - GLCM (Grey Level Co-occurrence Matrix)

  Autocorrelation, cluster prominence, cluster shade, cluster tendency, contrast, correlation, difference average, difference entropy, difference variance, inverse difference, inverse difference moment, inverse difference moment normalized, inverse difference normalized, information measure of correlation 1, information measure of correlation 2, inverse variance, joint average, joint entropy, joint energy, maximal correlation coefficient, maximum probability, sum average, sum entropy, sum squares;

  - GLRLM (Grey Level Run Length Matrix)

  GL non uniformity, GL non-uniformity normalized, GL variance, high GL run emphasis, long-run emphasis, long-run high GL emphasis, long run low GL emphasis, low GL emphasis, run entropy, run length non-uniformity, run length non-uniformity normalized, run percentage, run variance, short run emphasis, short run GL emphasis, short run low GL emphasis;

  - GLSZM (Gray Level Size Zone Matrix)

  GL non uniformity, GL non uniformity normalized, GL variance, high GL zone emphasis, large area emphasis, large area high GL emphasis, large area low GL emphasis, low GL zone emphasis, size zone non uniformity, size zone non uniformity normalized, small area emphasis, small area high GL emphasis, small area low GL emphasis, zone entropy, zone percentage, zone variance;

  - GLDM (Gray Level Dependence Matrix)

  Dependence entropy, dependence non uniformity, dependence non uniformity normalized, dependence variance, GL non uniformity, GL variance, high GL emphasis, large dependence emphasis, large dependence high GL emphasis, large dependence low GL emphasis, low GL emphasis, small dependence emphasis, small dependence high GL emphasis, small dependence low GL emphasis;

  - NGTDM (Neighboring Gray Tone Difference Matrix)

  Busyness, coarseness, complexity, contrast, strength.

Section S3: hyperparameters tuning

The hyperparameters tuning was performed through grid search in a repeated stratified 5-fold cross validation procedure, stratified over the label. TABLE S2 contains the range of possible values for each hyperparameter tuned.

Table S2. Possible values for each Hyperparameter during grid-search optimization.

**s-SVM**	α	0.0001, 0.001, 0.01, 0.1, 0.5, 1, 10, 100
	Rank ratio	1e-5, 0.0001, 0.001, 0.2, 0.5, 0.8, 1
	Optimizer	'avltree', 'rbtree', 'direct-count'
**r-Cox**	L1-ratio	1e-5, 1e-4, 0.001, 0.2, 0.5, 0.8, 1
	Penalty factor	1, 0.1

Section S4: s-SVM results

This section contains the results relative to s-SVM models in terms of Harrell C-index, as shown in Table S1. Results are lower than r-Cox performances, except for HD-LR predictions from DVH-based models, that anyway are not promising.

Table S3. Results of the s-SVM models with different settings. Results are shown in terms of median(IQR).

		**Dosiomics-based**			**DVH-based**		
	**Selection**	LET_d	$\mathbf{D_{MKM}}$	$\mathbf{D_{LEM}}$	LET_d	$\mathbf{D_{MKM}}$	$\mathbf{D_{LEM}}$
LC vs. LR	MW	0.69(0.22)	0.67(0.15)	n.a.	0.51(0.25)	0.63(0.15)	0.63(0.16)
	LASSO	0.65(0.19)	0.61(0.18)	0.63(0.18)			
LC vs. HD-LR	MW	0.70(0.23)	0.71(0.21)	0.77(0.21)	0.59(0.26)	0.56(0.21)	0.60(0.26)
	LASSO	0.68(0.25)	0.70(0.24)	0.75(0.26)			

Section S5: r-Cox results

LC vs. LR

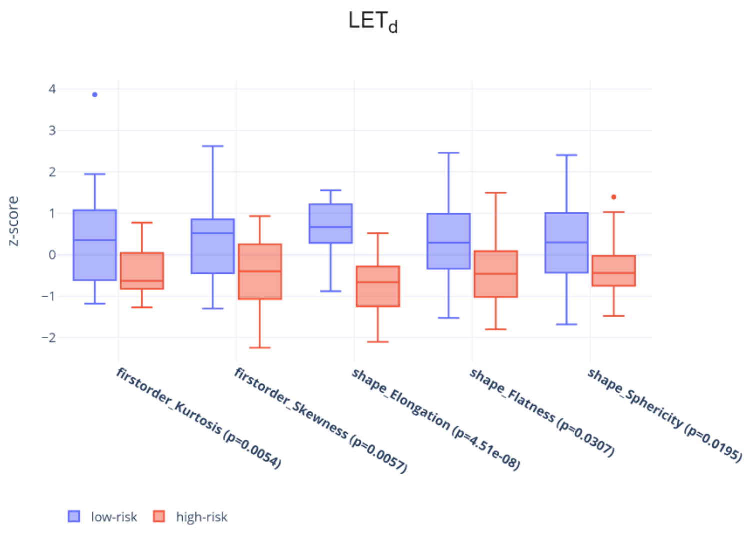

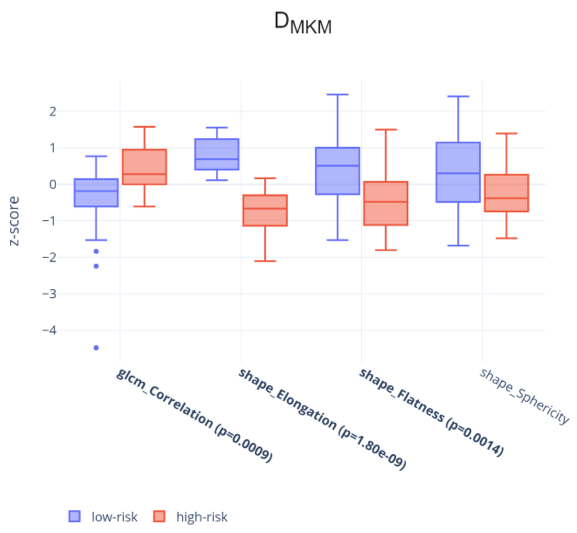

Figure S1. Z-standardized features as stratified according to the risk (high in red, low in blue) of showing a Local Recurrence (LR). The models are built with dosiomics features from LET$_d$ (left) and D$_{MKM}$ (right) maps, after MW feature selection.

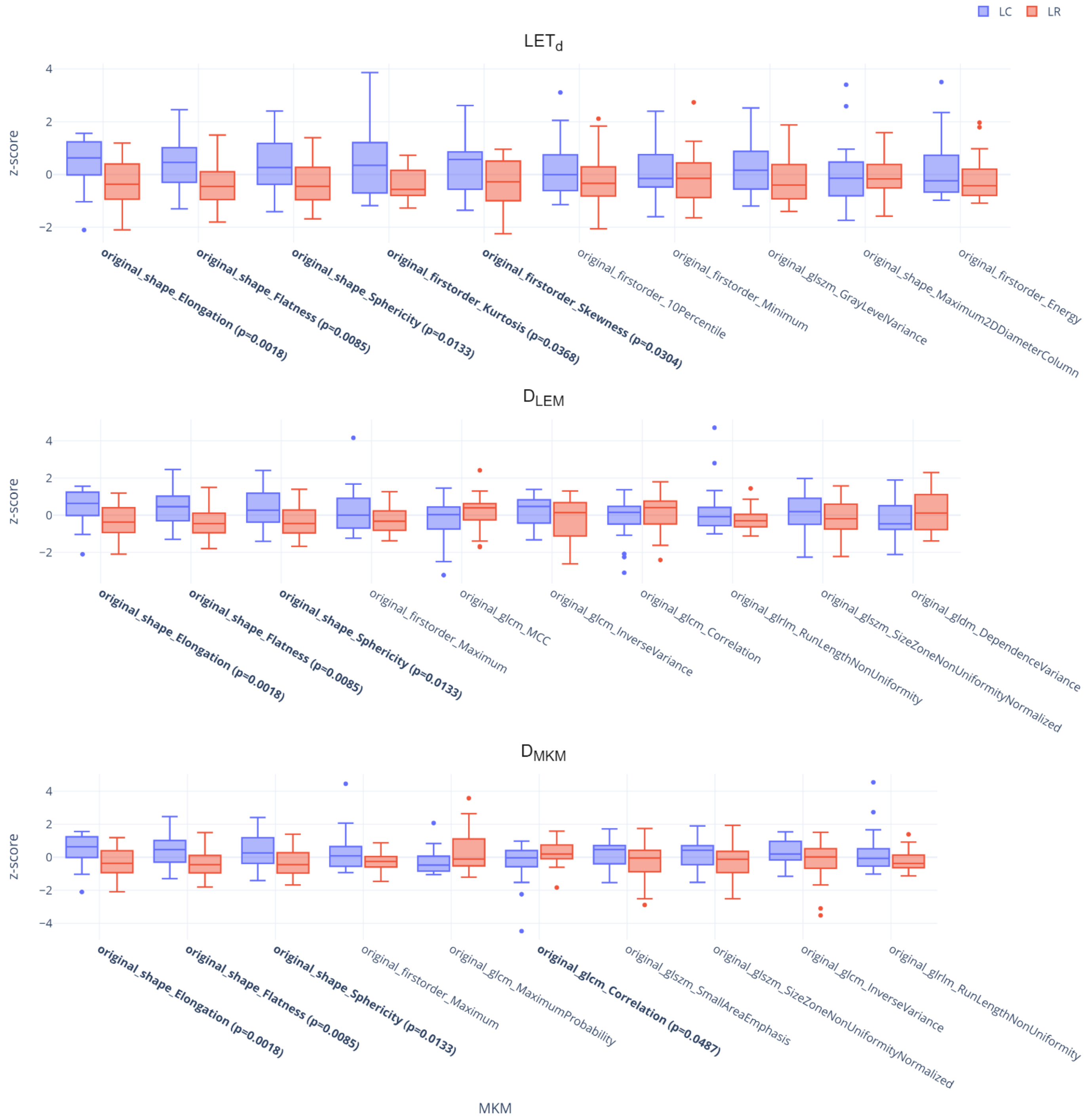

Figure S2. Z-standardized features as stratified according to the risk (high in red, low in blue) of showing a Local Recurrence (LR). The models are built with dosiomics features from LET$_d$, D$_{LEM}$ and D$_{MKM}$ (top to bottom) maps, after LASSO feature selection.

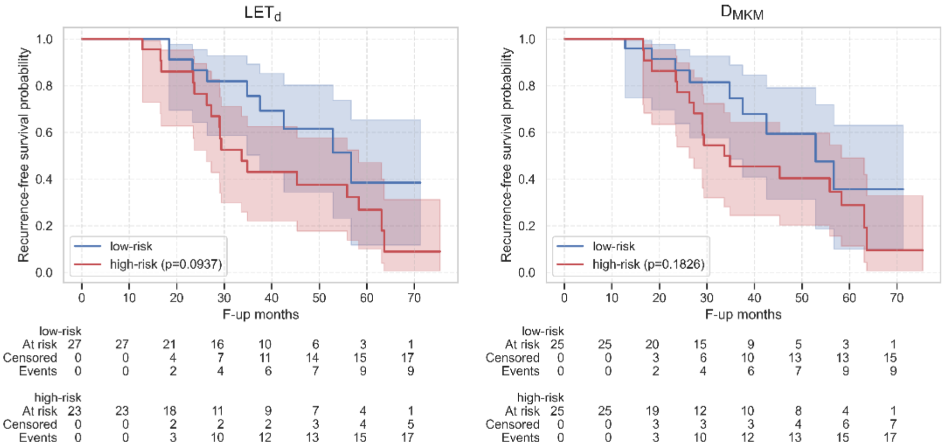

Figure S3. Kaplan-Meier survival curves for patients at high-(red) and low-risk (blue) of a LR as stratified by r-Cox using LETd (left), and DMKM (right) features after MW selection. Shaded areas show curves confidence intervals and the p-values obtained from log-rank test between high- and low- risk patients are reported in the legend. Below the plot, the number of patients belonging to each risk group at specific time points (months) is reported.

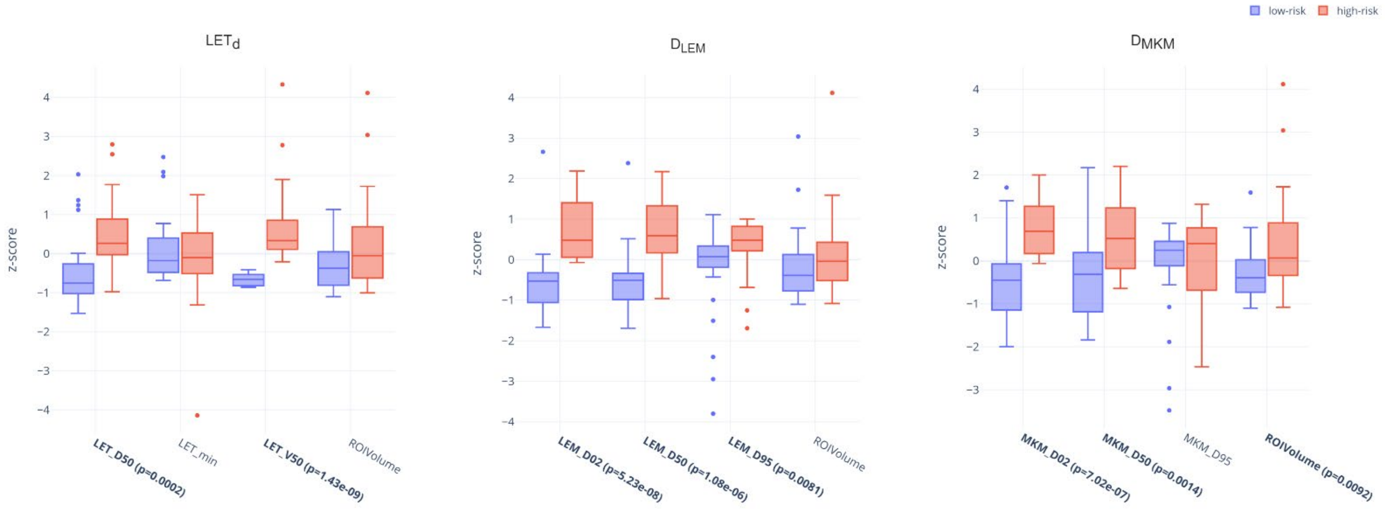

Figure S4. Z-standardized features as stratified according to the risk (high in red, low in blue) of showing a Local Recurrence (LR). The models are built with dosimetric (DVH-based) features from LETd, DLEM and DMKM (top to bottom) maps.

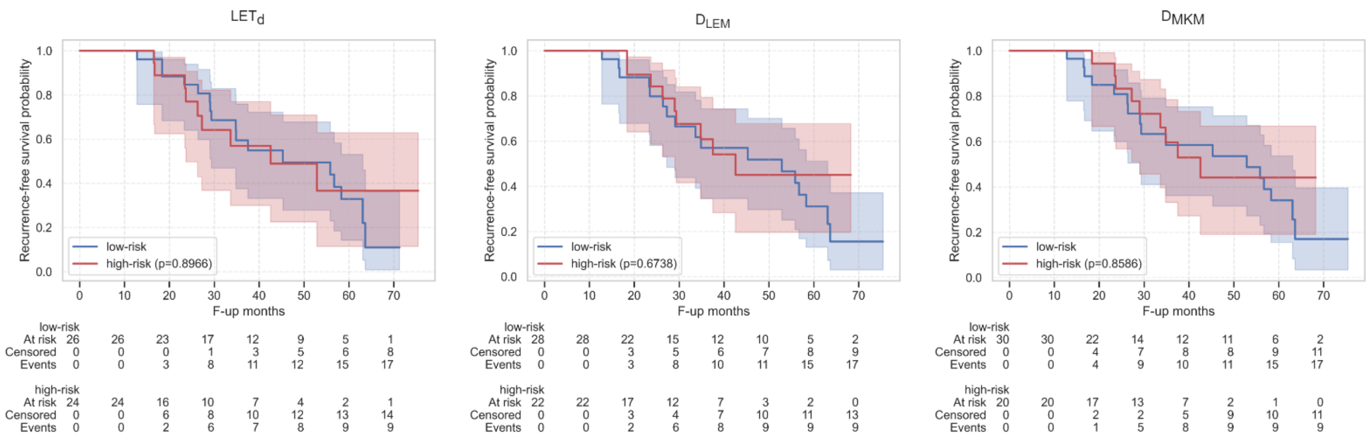

Figure S5. Kaplan-Meier survival curves for patients at high-(red) and low-risk (blue) of a LR as stratified by r-Cox using LET$_d$, D$_{LEM}$ and D$_{MKM}$ (left to right) dosimetric (DVH-based) features. Shaded areas show curves confidence intervals and the p-values obtained from log-rank test between high- and low- risk patients are reported in the legend. Below the plot, the number of patients belonging to each risk group at specific time points (months) is reported.

LC vs. HD-LR

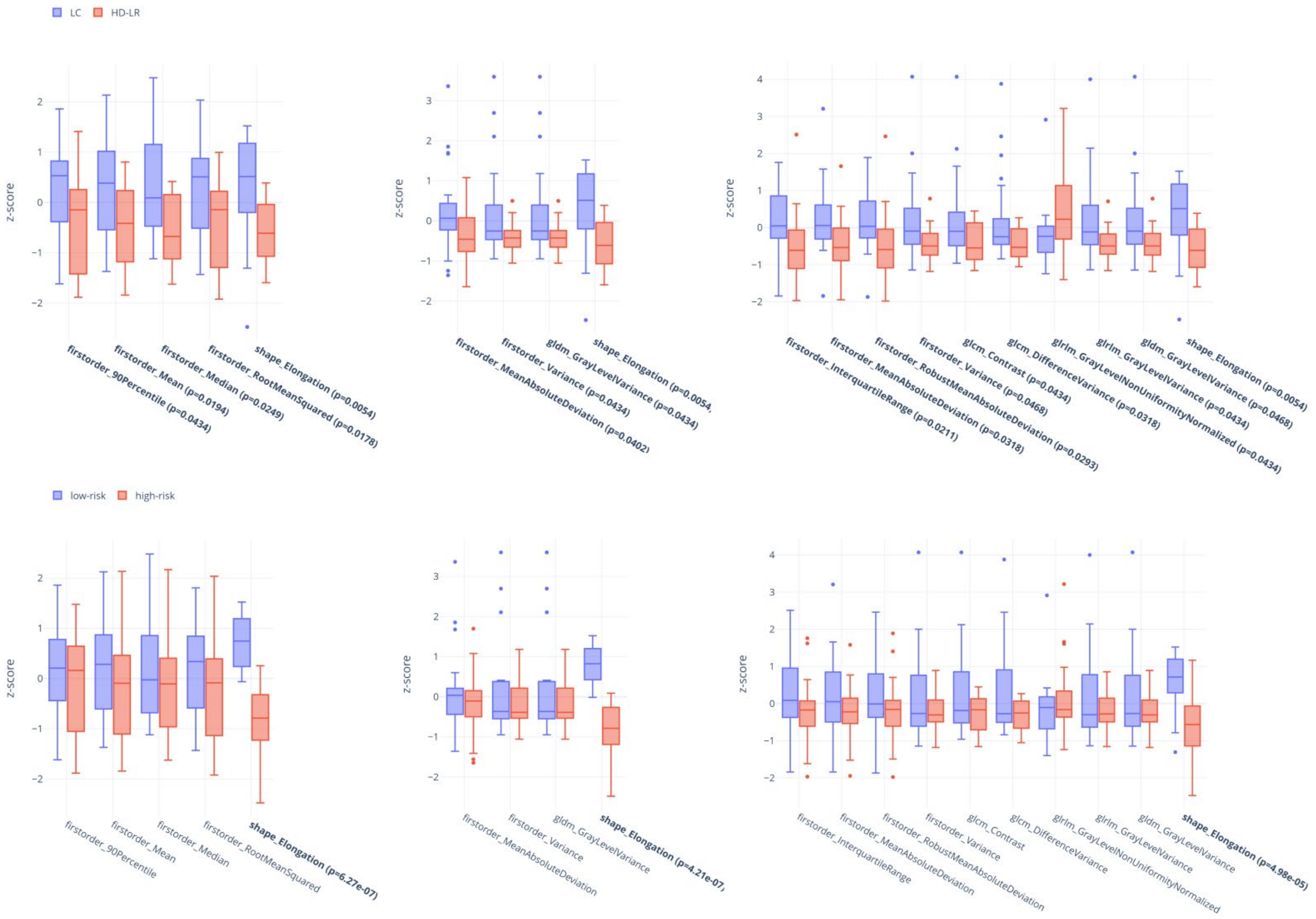

Figure S6. Z-standardized features as stratified according to the real class (First row; HD-LR in red, LC in blue) and the risk (Second row; high risk in red, low in blue) of showing a Local Recurrence in a High Dose region (HD-LR). The models are built with dosiomics features from LET$_d$, D$_{LEM}$ and D$_{MKM}$ (top to bottom) maps, after MW feature selection.

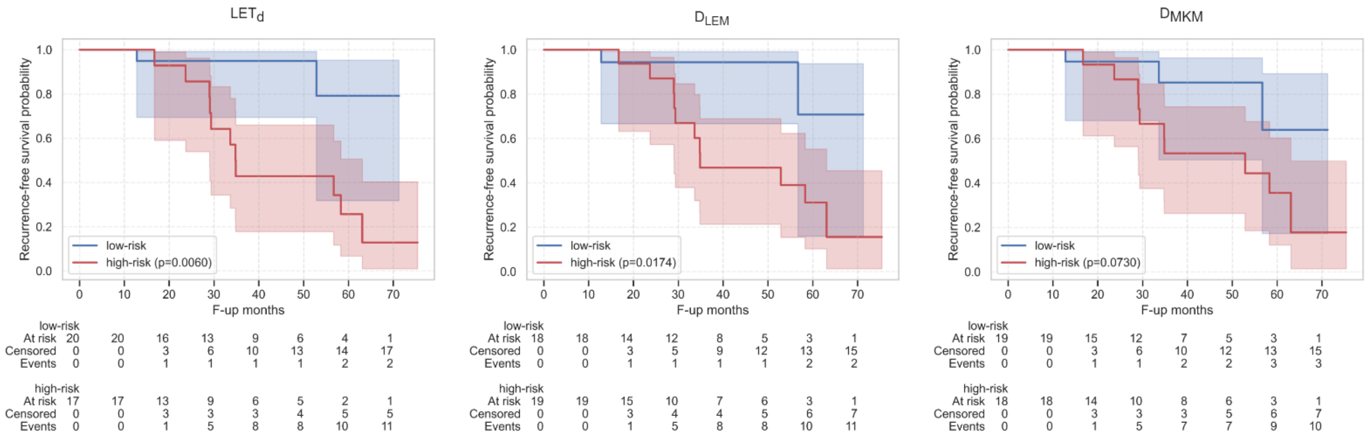

Figure S7. Kaplan-Meier survival curves for patients at high-(red) and low-risk (blue) of a HD-LR as stratified by r-Cox using LET_d, D_{LEM} and D_{MKM} (left to right) features after MW selection. Shaded areas show curves confidence intervals and the p-values obtained from log-rank test between high- and low- risk patients are reported in the legend. Below the plot, the number of patients belonging to each risk group at specific time points (months) is reported.

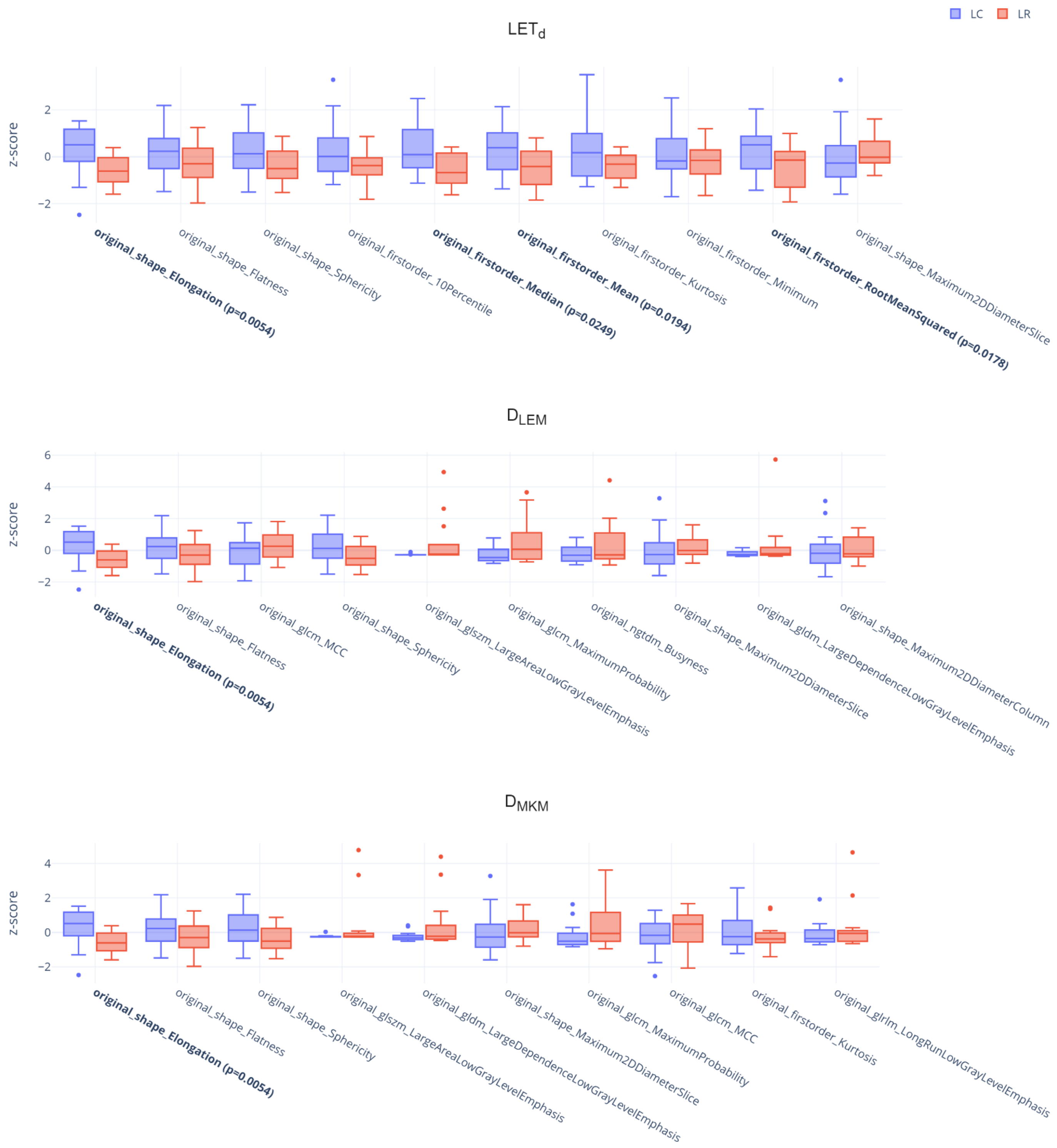

Figure S8. Z-standardized features as stratified according to the risk (high in red, low in blue) of showing a Local Recurrence in a High Dose region (HD-LR). The models are built with features from LET_d, D_{LEM} and D_{MKM} (top to bottom) maps, after LASSO feature selection.

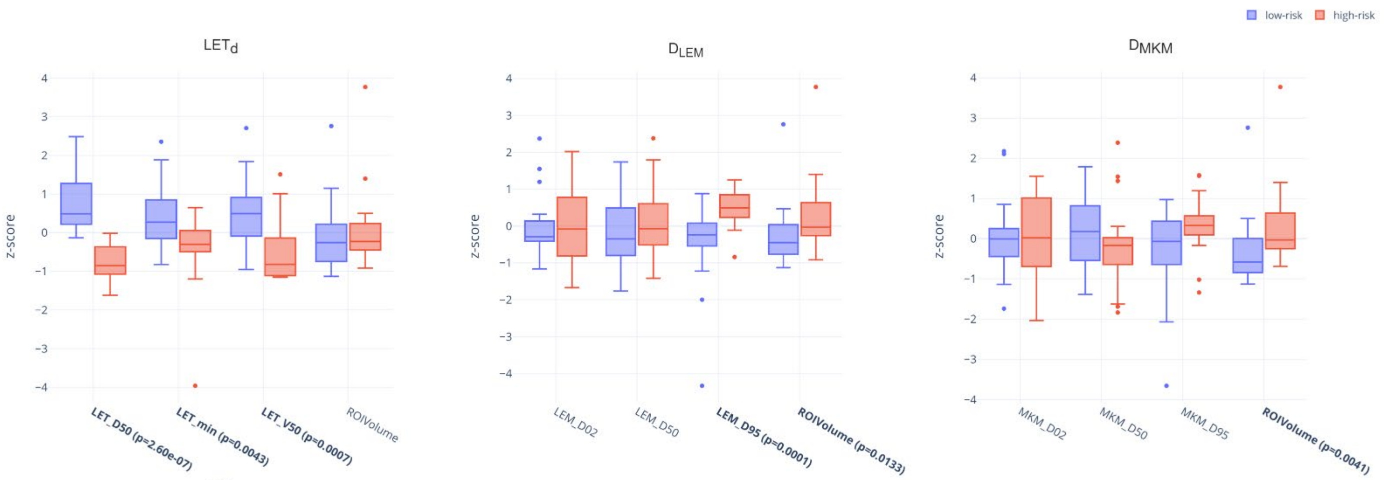

Figure S9. Z-standardized features as stratified according to the risk (high in red, low in blue) of showing a Local Recurrence in a High-Dose region (HD-LR). The models are built with dosimetric (DVH-based) features from LETd, D_{LEM} and D_{MKM} (left to right) maps.

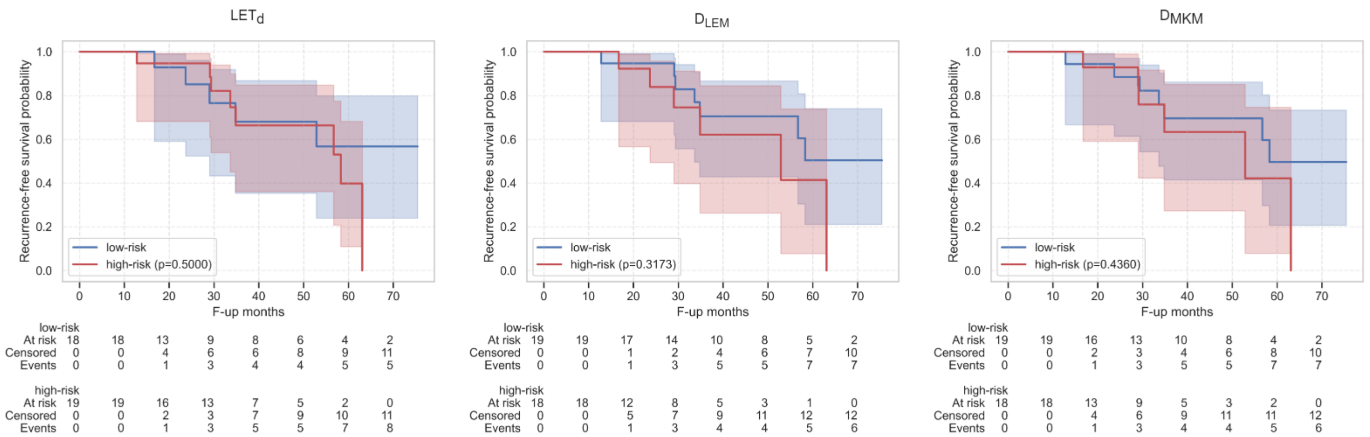

Figure S10. Kaplan-Meier survival curves for patients at high-(red) and low-risk (blue) of a HD-LR as stratified by r-Cox using LETd, D_{LEM} and D_{MKM} (left to right) dosimetric (DVH-based) features. Shaded areas show curves confidence intervals and the p-values obtained from log-rank test between high- and low- risk patients are reported in the legend. Below the plot, the number of patients belonging to each risk group at specific time points (months) is reported.

Section S6: Additional analyses: shape-based models

This model was trained to predict both LR (Figure S11, top) and HD-LR (Figure S11, bottom), following the same procedure reported in the manuscript. The median C-index (IQR) for LR prediction was 0.68 (0.18), while it reached 0.80 (0.42) for HD-LR, but no survival curve showed to be significantly separated, with p values of 0.055 and 0.051 for LR (Figure S12, left) and HD-LR (Figure S12, right), respectively. These results, which are aligned with the C-index from the other models, suggest that the sole shape features can discriminate quite efficiently the two classes, but the presence of first-order and texture feature can be relevant for the improvement of the overall performance, as a significative separation of survival curves. Despite the apparently good performance of shape features, these are subject to a high inter-observer variability in terms of target delineation [39], therefore predictions cannot be uniquely based on their values. However, Elongation, Flatness and Sphericity proved again to be the only features to be significantly different between high- and low-risk classes, confirming their relevant prognostic power.

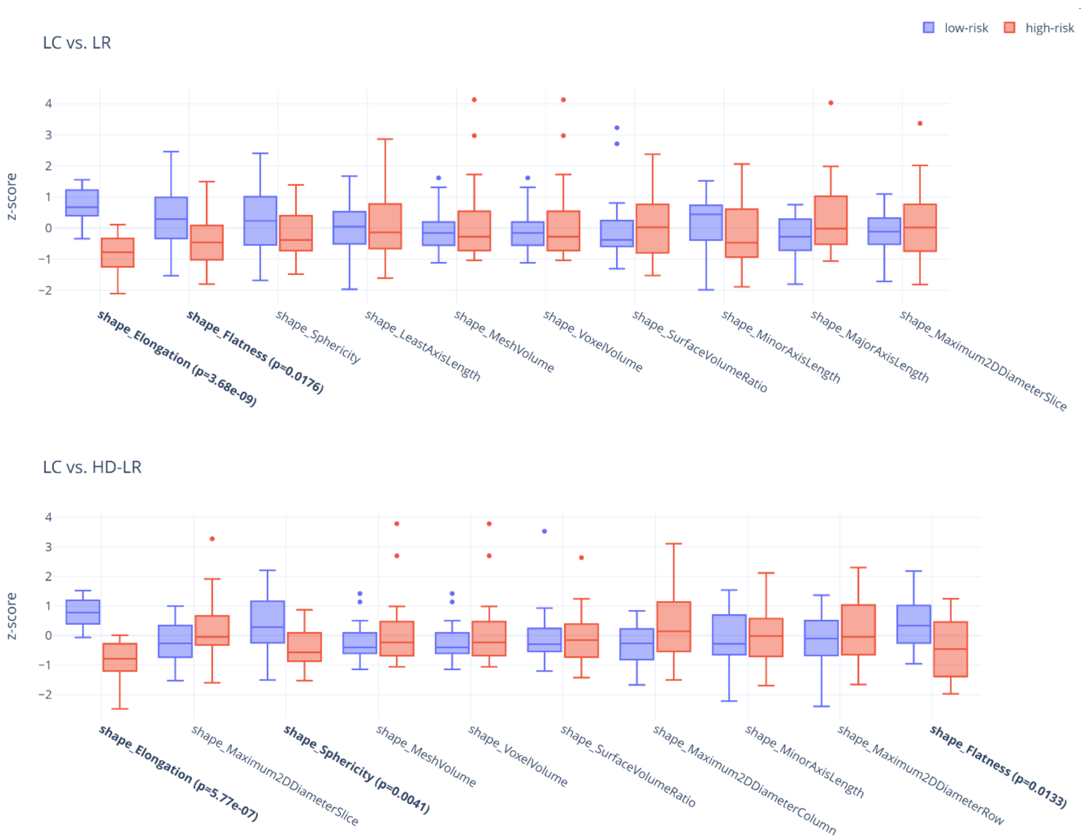

Figure S11. Z-standardized features as stratified according to the risk (high in red, low in blue) of showing a LR (top) or HD-LR (bottom). The models are built with LASSO-selected shape features.

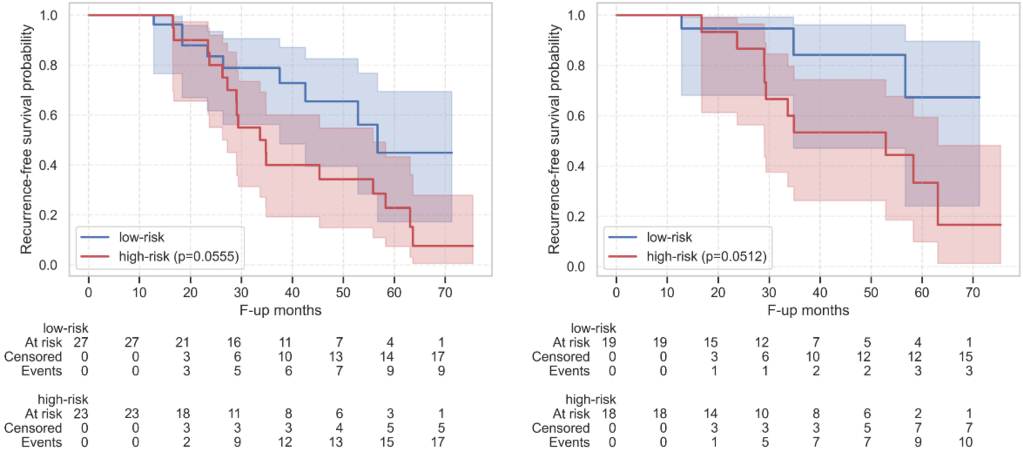

Figure S12. Kaplan-Meier survival curves for patients at high-(red) and low-risk (blue) of a LR (left) or HD-LR (right) as stratified by r-Cox using LASSO-selected shape features. Shaded areas show curves confidence intervals and the p-values obtained from log-rank test between high- and low-risk patients are reported in the legend. Below each plot, the number of patients belonging to each risk group at specific time points (months) is reported.
